# Ethnobotanical knowledge acquisition during daily chores: the firewood collection of pastoral Maasai girls in Southern Kenya

**DOI:** 10.1186/s13002-016-0131-x

**Published:** 2017-01-13

**Authors:** Xiaojie Tian

**Affiliations:** Graduate School of Asian and African Area Studies, Kyoto University, 46 Shimoadachi-cho, Yoshida, Sakyo-ku, Kyoto 606-8501 Japan

**Keywords:** Traditional ecological knowledge, Pastoral Maasai, Children, Daily practice, Adaptive knowledge acquisition

## Abstract

**Background:**

Researchers considering children’s traditional ecological knowledge (TEK) highlighted the importance of examining children’s daily activities as empirical contexts for its acquisition. Many of them evaluated children’s TEK acquisition linearly as gain or loss, and paid less attention to the adaptive nature of this knowledge system and the social relationships arising from its acquisition processes. This study approaches children’s TEK acquisition considering these abovementioned aspects. I utilize pastoral Maasai girls’ firewood collection as a case study, and analyze the personal, interpersonal and cultural institutional aspects of girls’ Ethnobotanical knowledge (EK) acquisition within this chore.

**Methods:**

Participant observation and unstructured interviews were used for data collection. I joined 12 girls (6 to 15 years old) on day trips for firewood collection, and documented their participation and performance during this chore. I observed interactions among girls and between girls and women concerning this activity, and investigated girls’ perceptions of local wood species via their descriptions. I also informally interviewed 15 women, between 20 and 80 years old on their evaluation of the wood species to be used as firewood.

**Results:**

Current diet change and gender-age roles in chore participation in Maasai society require females to continually participate in firewood collection. Within this social context, girls intensively participated in day trips of firewood collection during the long-term vacation in the dry season. They collected a sizable amount from 24 plant species, and generated EK through personal sensual experiences, such as fragrance, hardness, and heaviness of different wood species. They acquired local taxonomy and terminology of different wood species, and learned others preferences for wood species used as fuel through interpersonal communication. These personal and interpersonal aspects, together with current diet change and division of labor within gender-age roles in Maasai society, provide EK with multi-dimensional meanings in current subsistence strategies.

**Conclusions:**

Results of this study show that girls acquired EK with multi-dimensional meanings through daily firewood collection, which cannot be only evaluated in a linear manner. Future studies focused on children’s TEK acquisition should consider the personal, interpersonal, and cultural institutional aspects of this adaptive knowledge system and children’s roles within it.

## Background

Growing interest in Traditional Ecological Knowledge in recent decades highlighted the importance of this adaptive knowledge system in the fields of biodiversity conservation and sustainable resource uses [1–3]. Meanwhile, increased researchers criticized the gradual loss of this knowledge system [4–6]. For example, pastoralists in east Africa substantially depend on ethnobotanical knowledge (EK) of local vegetation to survive and feed their livestock through naming, classifying, and utilizing plants as fodder, medicine, construction materials, and fuel [7–10]. Researchers and policymakers have commended this EK for the alternative solutions it provides in local rangeland management and sustainable development [3, 11, 12]. However, other researchers focused on the problems of pastoralists’ TEK acquisition critically emphasized the loss of this knowledge among young generations [9, 13, 14], Most of these studies evaluated TEK acquisition linearly as gain or loss by individuals and paid little attention to its dynamic and adaptive nature [15, 16]. In contrast to the acquisition of knowledge in a school setting, TEK acquisition is heavily embedded in individuals’ daily activities in local social complexities [15, 17], and should not be evaluated purely on personal matters.

To grasp how indigenous peoples acquire ecological knowledge, including EK, researchers first looked at how it was unevenly distributed in local contexts. These studies stressed that gender roles and the life histories of individuals in a particular indigenous community influence the distribution of this knowledge [18–21]. Other researchers addressed the importance of capturing this knowledge within daily activities and applied quantitative approaches for understanding of the daily context within which children access and utilize natural resources in local ways [22–27]. However, as Ingold [28] emphasized, “practitioners’ environmental knowledge lies in skills that is in developmentally embodied capacities of awareness and response built up through a history of involvement with the land and its inhabitants” (p. 301). Therefore, a quantitative approach itself is insufficient for examining the systematic characteristics that influence individuals’ knowledge acquisition [29].

Situated learning theories have been developed in disciplines such as developmental psychology and cognitive anthropology. Psychologist Vygotsky [30] initiated sociocultural theory and argued how cultural beliefs and attitudes related to learning by individuals. As a result, many researchers sought effective approaches to understand the relationship of daily activities with human development in their sociocultural context [31–34]. Following on from sociocultural theory, Rogoff [32] contended the mutual integration between individuals’ development and cultural changes. She concluded that in order to understand human development within daily activities, it is necessary to focus on three levels of analysis and their correlations: the personal, interpersonal and cultural-institutional aspects. Sociocultural theory and the qualitative approaches developed for understanding learning behaviors have been widely used in studies concerning language acquisition and child development [34]. Recent studies concerning TEK acquisition among children paid attention to personal achievements [25, 26], however, with less consideration of the interpersonal, cultural-institutional and the correlation of these three aspects of this knowledge system. This study aims to understand the multi-dimensional factors that influence children’s TEK acquisition in their daily life. Taking firewood collection as the vital daily context for girls to access EK in current Maasai society, I analyze the multi-dimensional factors with regard to their personal, interpersonal and cultural-institutional aspects, and their correlations within this activity.

There are two main reasons why this study targets girls’ firewood collection in a pastoral Maasai community. First, this activity is one of the most important purely female 1 subsistence activities in pastoral Maasai society. It provides the best daily context for understanding Maasai girls’ EK acquisition. Second, significant lifestyle changes in Maasai society can be observed especially concerning local diet changes [35]. Firewood collection is one of the most vital daily strategies that female family members have continued and transformed for coping with current social changes [36]. The focus on girls’ firewood collection can contribute to a better understanding of EK acquisition as a local response by Maasai to concurrent external changes.

In the following part, I briefly introduce the study area and research methods. The findings of this study review the personal, interpersonal and cultural-institutional aspects of EK within girls’ daily firewood collection. Further discussions focus on analyzing the systematic characteristics of the described three aspects and their correlations in terms of Maasai children’s EK acquisition through this chore.

### Study area

**Fig. 1:**
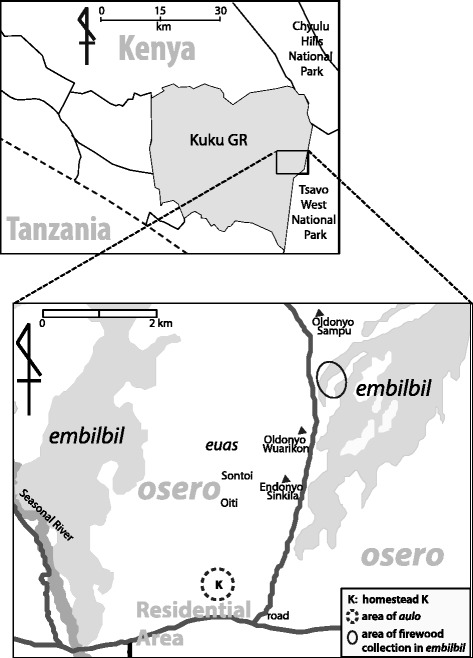
Location and landscape of the study area with local names

This study took place in a Maasai village in southern Kenya on the eastern side of the Kuku Group Ranch (KGR) (Fig. 1). The area has a semi-arid climate with two rainy seasons: March to May and November to December [37]. Dominant vegetation of KGR includes grassland, bushland, and dense forests on lava flows [37, 38]. *Acacia* and *Commiphora* are the main tree species in KGR [39]. Local people living in the study area indicated that they could easily access wood species that have multiple uses, such as *Acacia tortilis* and *Acacia mellifera*. There are two lava flows on the village’s eastern and western sides descending from the Chyulu Hills. According to local people, important wood species such as *Olea europaea* ssp. *cuspidata* and *Dombeya kirkii* can be easily found inside the lava flows in this area.

A drought in 2009 led to the loss of more than 80% of the livestock in Kuku GR [40]. Local Maasai have a strong desire to replenish their livestock and this situation contributed to the increased involvement of local Maasai in various economic activities, both within and outside the area [39]. Children increasingly participate in formal education, and according to the two local chiefs, more than 80% of the village children of primary school age attended local school in 2015. Influenced by diversified economic activities, the diet of local Maasai has gradually shifted from being milk and meat centered to more dependence on agricultural products [39]. Along with these changes, firewood has become an increasingly critical resource to support local energy use.

Historically, local Maasai participate in subsistence related activities according to their gender and age [41]. Apart from adults, children play significant roles in daily subsistence [41, 42]. Children (*inkera*) in Maasai society, refer to newborn persons up until they receive circumcision. In recent decades, the act of circumcising girls has declined. Local Maasai treat females who are not yet married as girls. Boys (*olaiyiok,* pl*. ilayiok*) participate in daily livestock grazing from an early age. When they become more skilled, they are responsible for both short and long distance grazing. Girls (*entoyie,* pl*. intoyie*) are mostly involved in daily chores related to preparing and distributing food, cooking, cleaning, and gathering firewood. They also participate in daily livestock tending, mainly through milking and watching livestock in and around their homesteads. Although children gradually spend more time at school, they still actively help tending livestock, and perform other chores during free time [42].

## Methods

The main methods of this study included participant observation and informal interviews in 2013 and 2015. Initially, my goal was to obtain a general understanding of Maasai children’s daily lives. I found Maasai girls participated in collecting firewood on the weekends during term time. I further informally interviewed school children in a local primary school with the help of a local research assistant who translated the local language. All girls who took part in the survey (*n* = 50, from 9 to 16 years old) said they gathered firewood during vacation.

Based on these findings, I conducted qualitative data collection during the long-term school vacation in the dry season, from the middle of August to the beginning of October 2015. For a total of 55 days, I focused on 12 girls aged 6 to 15 years old, and participated in their daily firewood collection. Seven of them (Nk, Tn, Lst, Ln, Sk, Tt and Ml) live in the same homestead (K), while the others (Mg, Chl, Rl, N, and Li) are from three different homesteads nearby. The girls from homestead K sometimes separated into two groups; one consisted of Nk, Tn, and Ml (Nk’s group), and others in Ln’s group. Each group had members from nearby homesteads. Since each group went to different locations to gather firewood, I mainly focused on the girls in Ln’s group.

During the 55 days of participant observation, I documented 21 day trips of firewood collection and recorded details of the participants, local names of wood species, and the weight of firewood that each participant collected on each trip. These data are listed in Table 2. The number of wood species collected by each girl, for every trip, are listed in Table 3. With this data and the times of each girl’s firewood collection, I calculated the collecting frequency of each girl on different wood species in Table 4. Since I mainly observed Ln’s group, the total number of trips that Nk, Tn, and Ml took part in and the amount of firewood they gathered are considered greater than the amount shown in Table 2. After the girls carried their loads home, I recorded the weight of firewood consumed in Ln’s household 2 within a 9-day period.

During participant observation, I recorded the locations of these trips with GPS, along with local names. After observing the process of collection and discussing firewood with different girls several times, I realized the importance of their physical senses in acquiring EK while collecting and utilizing firewood. For instance, they described the differences in wood species such as the smell, the hardness and heaviness of the wood, as well as information on how they use machetes to cut the wood pieces. Thus, I considered individual girls’ sensory perceptions such as how they chose, cut, and packed firewood for 10 out of the 21 day trips.

I conducted informal interviews with 15 women from 20 to 80 years old, about their assessment of the firewood the girls collected. I asked them to explain whether the wood species were good for firewood and why. These data were summarized in Table 1 together with information regarding voucher specimens. Voucher specimens were identified and deposited in the Botany Department of the National Museum of Kenya.

## Results

The results are presented in three parts. I firstly focus on cultural institutional aspects related to firewood collection by reviewing the geographical area for firewood collection and use in current Maasai society, together with generally shared discernment of firewood among local females. I then focus on individual girls’ participation in this chore via their household contribution, and their ways of valuating and collecting the firewood. In the last part, I focus on the interpersonal aspects related to this chore through reviewing the information sharing, communications and other daily interactions among girls, as well as between girls and adults.

### Geographical space for firewood collection and use

Local Maasai build fences around their homesteads using thorn branches from acacia trees. Female stock and use firewood in different living spaces related to other daily activities such as livestock management. In a homestead, small houses built for human use surround livestock huts. The kitchen and sleeping area consist of one room inside the house. In the kitchen area, half of the big wooden shelves usually provide room for utensils; the rest is used to store firewood. A three-stone hearth in the center serves as a cooking area. In the backyard, women and girls build temporary square wooden stand to store firewood during the rainy season. It is built approximately 30 cm above the ground in order to keep the wood dry, and big crude sticks at each corner prevent the wood from collapsing. Sometimes more than one of these structures can be found in a single household. Older girls who can carry heavy loads of firewood make their own structures when competing with their mothers.

Females usually collect firewood from different places. Surrounding each homestead (*enkang*), an area of approximately 90,000 m^2^, called the *aulo*, is set aside for grazing the homestead’s juvenile livestock. It is also an area that children under five usually perform their daily tasks such as firewood collection. Non-residential areas outside the *aulo* are generally called *osero*, the bush. Except for small children, who are not allowed to venture out into the *osero* alone due to dangerous wildlife, older children and adults go off in different directions to graze livestock, collect plants, and undertake other subsistence-related activities. At homestead K, people usually go to the eastern side of the village toward Chyulu Hills to herd and gather firewood.

In *osero*, numerous local words are used to describe the land and certain locations (Fig. 1). Some places were named after local wood species. For example, the place called Oiti is derived from the *Acacia mellifera*. Some place names refer to special events that either happened or are mentioned in mythical stories. Sontoi is a place name derived from a person who used to cook milk tea under the trees when returning from collecting medicinal plants in the Chyulu Hills. Names are also often mixed to describe a certain location. For instance, girls from homestead K said they went to Euas Oldonyo Wuarikon to gather firewood. It means they went to the plain area (*euas*) located near the mountain Oldonyo Wuarikon.

Girls start collecting firewood from an early age. At 3 years old, they collect small sticks from the fence around the homestead, or under trees in the *aulo*. They tie sticks together with rope ties and put the tiny load on their back. After taking the load home, older girls or mothers sometimes used these small sticks for real cooking. Girls become familiar with different species of wood initially in the *aulo* before they travel longer distances for gathering. They collect dried wood for cooking, and listen to mothers and older girls talking about firewood, the location for collecting, and fuel efficiency of different wood species. When they start to gather firewood from further places at approximately 6 years old, they can describe a considerable number of species’ names and the sites where these species can be found.

### Females’ discernment of wood species

During day trips for firewood collection, the girls in homestead K collected a total of 24 different wood species for firewood use (Table 1). In this section, I explain females’ perceptions of these wood species for fuel use. Women also use other wood species as firewood in daily life, however, in order to compare girls and women, I only focus on these 24 types in this study.

**Table 1:** Names of collected fuelwood species and comments on their heating efficiency

No.***	Local name of the species	Scientific name	Location	Life form	Heating efficiency	Smoke	Other features
4	oiti	*Acacia mellifera* (Vahl) Benth.	a	tree	good	no	good charcoal
2	olkiloriti	*Acacia nilotica* (L.) Willd. ex Delile	b	tree	good	no	heavy
8	enkilelio	*Acacia senegal* (L.) Wild	b	tree	*	*	easy to break into pieces, it does not stay in the fire for a long time
24	olderkesi	*Acacia senegal* (L.) Wild	c	tree	*	a lot	leaves ashes after being burnt
3	oltepesi	*Acacia tortilis* (Forssk.) Hayne	a	tree	good	*	hard, leaves ashes after being burnt
19	osalagi	*Balanites aegyptiaca* (L.) Delile	a	tree	*	a lot	the smoke hurts one’s eyes
21	olng'osua	*Balanites glabra* Mildbr. & Schltr.	a	tree	good	a lot	the smoke hurts one’s eyes and has a bitter taste when floats into mouth
6	nenkopang	*Bridelia taitensis* Vatke & Pax	b	shrub	*	a little	too small for use as firewood
13	enkonerei	*Commiphora schimperi* (O.Berg) Engl.	b	tree	*	*	
17	osioki	*Cordia monoica* Roxb.	b	tree	*	yes	the smoke has a good smell, the dried wood easily attracts ants
14	oltiasika	*Dalbergia melanoxylon* Guill. & Perr.	b	tree	good	no	
15	olalejani	*Dodonaea viscosa* (L.) Jacq.	b	shrub	good	a little	the smoke has a bad smell
25	olporokuai	*Dombeya kirkii* Mast.	c	shrub	good	no	it can stay in the fire for a long time
9	esamantet	*Grewia fallax* K.Schum.	a	tree	*	no	it cannot stay in the fire for a long time
5	esiteti	*Grewia* sp.	b	tree	*	no	
12	olmangulai	*Grewia villosa* Willd.	b	shrub	*	yes	too small for use as firewood
16	olorien	*Olea europaea* ssp. *cuspidata* (Wall. & G.Don) Cif.	c	tree	*	yes	the smoke has a good smell, and is used to clean milk containers
7	enkitarrae	*Opilia amentacea* Roxb.	c	liana	*	no	too small for use as firewood
1	enkorsiyanchoi	*Ormocarpum kirkii* S.Moore	b	tree	good	no	heavy, good charcoal
11	olokunonoi	*Ozoroa insignis* Delile	b	tree	*	no	the wood has a bad smell
18	oltimigomi	*Pappea capensis* Eckl. & Zeyh.	c	tree	*	yes	the smoke has a good smell, for use as tea leaves
10	olmisigiyoi	*Rhus natalensis* Krauss	b	shrub	*	no	good charcoal, it can stay in the fire for a long time
22	entulele**	*Solanum incanum* L.	a	grass	*	no	it burns out easily
23	olegipeta	*Teclea simplicifolia* (Engl.) Verd.	c	tree	*	*	

Notes: data was collected from interviews with 15 women (who ranged in age from their 20s to 80s), and listed with alphabet order of the scientific names. Diverse answers can be found concerning the described features of wood species, which are marked with *. Because this paper focuses on the EK acquisition of girls, details of the differences in women’s descriptions and their reasons would not have been discussed. Location: a-Oiti, b-Oldonyo Wuarikon, c-Embilbil Oldonyo Sampu (see Fig. 1)

**Due to inefficient voucher information, I could not get identification information of this plant species. I adopted its scientific name from Kiringe’s study [39]

***No. refers to the voucher numbers that provided for plant identification

Local females choose different species considering various factors related to collecting and cooking. These features include accessibility of different species, heaviness of the wood, wood condition (such as dryness and weathering), and performance of the wood while burning (such as fuel efficiency, the amount of smoke, and the ashes and charcoal it produces after burning). Every wood species has been mentioned by local females as good in some respects for use as firewood in regard to its performance, and personal preferences when cooking different foods (Table 2). For instance, some women described *Solanum incanum* as not suitable for firewood because it burns out quickly; however, other women recommended using it to prepare milk tea for guests that suddenly drop by for a visit.

**Table 2:**
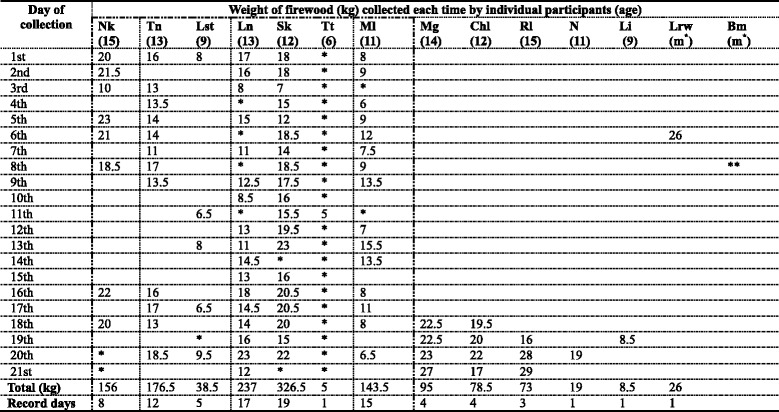
Detailed information on 21 day-trips for firewood collection, with 12 girl participants

Notes: Participants marked by dashed line live in the same homestead (homestead K). Children of different households are separated by dotted lines. Blank spaces mean that no record was made that day

*: Indicates a girl did not collect firewood on that day. **: Bm was confirmed going firewood collection, but the amount of her load was not weighted. m^*^: Married women who join the children in collecting firewood

Regarding some species, local females voiced different opinions on fuel efficiency and the amount of smoke. They said that aside from differences among species, various natural features of wood (such as the availability of bark, dryness and weathering) vary a lot from one piece to another, even within the same species. They said that completely dry wood from a preferred wood species is suitable for use as firewood. Girls also explained: “Choosing pieces of wood to take from a tree [for use] as firewood is like gathering fruit. You take the ripe pieces and leave the unripe ones.” Before cutting a branch, they usually cut a small piece of skin from it to check whether it still has a green color and moisture under the bark. This way of choosing possibly contributes to the long-term survival of the trees as well as long-term availability of firewood.

Local females indicated that the more weathered parts the wood has, the more smoke it produces. They mentioned a tree’s bark and roots as causes of smoke. However, the smoke’s properties (which the bark and roots produce during burning) differ depending on the species. For instance, the women said that wood from *Acacia mellifera* has no smoke and no smell, whereas *Acacia tortilis* produces a lot of smoke especially when the piece of wood has bark. The fragrance comes from the smoke can sometimes be favorable. Compared with smoke from *Acacia tortilis*, described as being able to make people’s eyes sting until they cry, *Olea europaea* ssp. *cuspidata* is highly valued as the smoke from this species has a sweet aroma. It is commonly used to clean milk containers in the local area. Similar to *Olea europaea* ssp. *cuspidata, Pappea capensis* also has a pleasant odor, and its bark is commonly used for tea.

### Tools and preparing wood for fuel use

Females use different tools to gather firewood in the study area. Two tools are used for cutting: the machete and the axe. According to the number of older girls and women in each household, more than one machete can be found in a household. When girls collect firewood, they are allowed to carry a machete, but never use an axe. Women only use axes when they cut a big trunk into smaller pieces.

Females use a belt to pack up wood, and a handmade cushion (*oltukus*) to put between their backbone and load in order to reduce the physical burden. The belt is usually made of cattle skin about 4.5 m long and 3 cm wide*.* There are shorter belts made from other materials, such as rubber, for small girls only. Girls usually take a coat or old, worn sweater with which to make the cushion (*oltukus*) on the way. Small girls who prefer to put the load on their head usually put *oltukus* between their head and the load. Mothers sometimes remind girls to take *oltukus* with them and to be careful of their backbone. Female Maasai also use these tools for other chores. For instance, the same belt is used to carry water. Women also use the belt along with a machete to collect grass and wood for house construction, as well as for amassing fodder for young and sick livestock at home.

When collecting and cutting, local females usually perform five different actions to convert wood into firewood:

1. *Agel*, which means to choose or sort, and refers to breaking a piece of wood using one’s bare hands.
2. *Adung* means cutting a piece of wood, a limb, or branches from a trunk using tools. Good performance of this action depends on whether the machete or axe can deeply hit the same end of the wood.
3. *Adany* refers to splitting a piece of wood into small pieces, especially when using an axe. Big pieces of wood from a tree’s limbs and trunk are usually cut into smaller sizes for cooking. Females perform this action during the trip, and also in the backyard before they use firewood for cooking.
4. *Aiper* means splitting wood into two pieces of the same size. It is different from *adany* because on most occasions, women describe this action as separating branches that divide in two different directions.
5. After cutting wood from a tree, females remove the thorns and small branches using a machete; this action is called *asul*.

It is worth noting that there are no standard ways of practicing these five actions when females collect the firewood. Rather, these actions are practiced in different ways among individuals with regard to personal habits and physical body features (e.g., height, strength and the preferred gestures of using the tools). The following section focuses on detailed descriptions of the girls’ personal contribution and participation in this chore.

### Girls’ household contribution through firewood collection

I identified how the girls participate in day trips, including information about the time required for this chore and the girls’ contribution to their households’ subsistence. Girls never go on day trips alone but as part of groups of two to nine girls, either family members or friends. I rarely saw women join girls’ groups during these excursions. During the survey, only two married women joined two different outings. According to local women, this is due to different walking speeds, and the fact that both children’s and adults’ different cutting and collecting skills would influence the work efficiency of each group. From the girls’ perspective, they prefer to go with other girls, with whom they share much in common. On the way, they also meet other groups of girls and women. Sometimes they change their route and continue the journey together.

Girls use a considerable amount of time during a day trip for firewood collection. Going, collecting, cutting, packing, and returning require about 3.5 h. Generally, girls spend about one hour travelling, and another 2.5 h collecting and cutting. Within the 2.5 h, there is also time spent on resting (approximately 10 to 20 min) and packing up the wood (approximately 20 to 50 min). It is very rare to observe the girls take a rest on the way.

Places that girls in homestead K visited the most frequently include Oiti, Euas Oldonyo Wuarikon, and Embilbil Oldonyo Sampu (Fig. 1). Nk’s group went to Embilbil Oldonyo Sampu more frequently, where hardwood species with good quality, such as *Dombeya kirkii* and *Olea europaea* ssp. *cuspidata*, can be found. In contrast, Ln’s group preferred to go to places relatively near the homestead, where various wood species exist. For instance, in Euas Oldonyo Wuarikon, dried *Ormocarpum kirkii*, *Acacia senegal*, *Dalbergia melanoxylon*, and many other species can be easily found during the dry season within a 3 km distance from homestead K.

Over the 21 day trips, the girls amassed a substantial amount of firewood (5 kg to 29 kg per day per girl), and seven girls from homestead K gathered a total of 1083 kg (Table 2). According to the quantity of firewood collected by each girl of a different age, I found a clear difference between girls younger than 11 years old and above. Girls at age 11 or older collected a comparable amount and quality of firewood similar to women.

Although the amount of firewood consumed differs based on household size, the total wood collected by girls in Ln’s household for example, was more than enough to supply their own household needs. In Ln’s household, there are eight family members. In 9 days, they consumed a total weight of 62.1 kg firewood for cooking and 25.5 kg for giving to others as gifts. Firewood that Ln and Sk collected during the 21 days (a total of 563.5 kg) could supply approximately 80 days of fuel in their household (Ln and Sk live in the same household, mean consumption of their household were recorded as 7 kg of firewood per day).

When I asked girls why they frequently fetched firewood and stocked a considerable amount at home, they said they wanted to prepare for the rainy season. There were also girls who told that they wanted to reduce their mothers’ burdens after they started school, and they expressed: “I feel better going and doing something, rather than staying at home doing nothing.”

### Individual girls’ performance during firewood collection

#### Younger girls

During the period of the survey, 6 years old Tt participated in one trip for firewood collection, and 9 years old Lst took part in five trips. Tt reached the place of Oiti (Fig. 1), about 2 km away from the homestead, and Lst reached Oldonyo Wuarikon, about 3 km away. Most of the firewood they gathered was in a weathered condition.

While collecting, they used a machete in different ways through trial and error. Within 65 min, Lst tried to cut (*adung*) seven branches from different trees, and only three times she obtained small pieces of firewood. During the same trip, Tt tried cutting (*adung*) two branches from *Acacia tortilis*, however, without removing any pieces from the tree. The two girls frequently cut off a small piece of bark and check the degree of wetness, even on pieces they had picked up from the ground. They frequently scratched off (*asul*) thorns and twigs from the wood in order to pack it up. In total, Lst’s load contained 15 different wood species, and Tt’s contained three. Rather than referring to the wood species, these two girls tended to select wood according to which pieces were driest. They could not completely discern the names of the wood they collected. Throughout the process, they repeatedly confirmed the species’ name and dryness of wood with each other. After taking the wood home, they sometimes confirmed the names of wood species again with their older sisters or mothers. They did this not because they do not know these species by their local names, but rather because it is sometimes difficult to match the names with the pieces of wood they collected, which come in a myriad of shapes and weathered conditions.

In total, Lst collected 38.5 kg during her five trips, and Tt gathered approximately 5 kg during the one trip she took. Technically, the two girls were not familiar with using a machete through *aiper* and *adany*; neither of them made efficient cuts during *adung*. However, rather than go home empty-handed, they gathered a considerable amount of firewood. Their physical condition limited the wood they could access - which reflected more complex conditions (e.g. weathering and separation) than the wood collected by older girls. While collecting, they developed their physical capabilities along with their skills to distinguish and collect different types of fuelwood.

#### Older girls

With regard to the ability to use tools and identify plants, girls older than 11 are physically more competent, skilled in collection and able to focus more on species they desire. Table 3 shows the numbers of wood species each girl collected during the 21 days of recorded trips. On every outing, the girls obtained different numbers of wood species. Compared with the younger girls, some older girls collected wood from only one plant species during one trip (Table 3). In fact, this is more difficult than choosing dried branches from different types of trees. For example, on one trip, Mg collected 28.5 kg from an *Ormocarpum kirkii* approximately two meters high. Unlike other species, this tree’s branches and twigs are never straight, but rather crisscross each other thickly, make it difficult to cut. Initially, Mg cut the biggest branches near the bole (*adung*). Leaving a small part of the branch connected to the bole, she dragged the big branch down (*ager*). She further cut and separated (*aiper*) the limbs from the big branch into preferred sizes on the ground. She then removed (*asul*) the smallest twigs and thorns that could not be used as firewood. In total, she packed 22.5 kg for herself, and left the remainder to the other girls.

**Table 3:** Number of wood species collected by informants during firewood collection

Day	Number of collected wood species by each informant (name with age)										
	Nk (15)	Rl (15)	Mg (14)	Chl (12)	Tn (13)	Ln (13)	Sk (12)	Ml (11)	Lst (9)	Li (9)	Tt (6)
1st	3				6	5	9	6	9		
2nd	4					2	4	4			
3rd	3				3	3	1^b^				
4th					3		3	2			
5th	2				3	3	4	5			
6th	4				3		3	4			
7th					2	4	5	5			
8th	*				1^b^	2	2	3			
9th					1^b^	2	4	2			
10th						2	4				
11th							5		3		3
12th						1^b^	3	3			
13th						1^b^	3	3	5		
14th						2		3			
15th						2	5		2**		
16th	2				2	2	5	2			
17th					4	1^b^	3	3	4		
18th	5	*	1 ^a^	4	6	*	5	6	*		
19th	6**	2	3	4	7**	2	1^a^		6**	3	
20th		6	3	3	7	*	*	5	4		
21st		3	5	3		4					

N (11) was not listed in this table due to lack of wood species information that she collected

Data from the same 21 days of firewood collection, as listed in Table 2

^a^: *enkorsiyanchoi* (*Ormocarpum kirkii* S.Moore) ^b^: *oiti* (*Acacia mellifera* (Vahl) Benth.)

*: the number of wood species was not sufficiently documented

**: only numbers of wood species had been recorded, however, the total amount of collected firewood of the participant during that day was not able to documented, thus had not show in Table 2

Unlike Mg, Sk preferred to collect different types of wood every time she went to gather wood. She liked wood with a good smell and color, for instance *Rhus natalensis.* She recommended this species due to its pink color and pleasant aroma. Every time that she cut down (*adung*) a branch of this species, she always split (*aiper*) it down the middle, and enjoyed its aroma. Different from Mg and Sk, Nk said she preferred to gather wood from lava areas (*embilbil*) somewhere farther away*.* Nk said that she liked wood with a pure white color, as well as hard wood, which is not as common and difficult to find. She explained that wood with a pure white color meant the wood was dry, with less weathering. She wanted to make her wood stand out of the best wood. In Table 4, the wood species frequently collected by Nk are among those that local people described as having good fuel efficiency, such as *Acacia mellifera*, *Teclea simplicifolia* and *Acacia nilotica*.

**Table 4:** Wood collection by 11 girl participants* with reference to the different plant species

No.***	Collected plant species**	Times (frequency-*i*) of each informant’s (including age) wood collection											Total times (frequency*-t*)	No. of people
		Nk (15)	Rl (15)	Mg (14)	Tn (13)	Ln (13)	Chl (12)	Sk (12)	Ml (11)	Lst (9)	Li (9)	Tt (6)		
4	*Acacia mellifera* (Vahl) Benth.	7 (0.88)	2 (0.5)	1 (0.25)	13 (1.08)	8 (0.47)	1 (0.25)	12 (0.63)	13 (0.87)	5 (0.83)			62 (0.69)	9
2	*Acacia nilotica* (L.) Willd. ex Delile	4 (0.5)		1 (0.25)	6 (0.5)		2 (0.5)	5 (0.26)	3 (0.2)	3 (0.5)			24 (0.27)	7
8	*Acacia senegal* (L.) Wild	2 (0.25)	2 (0.5)	1 (0.25)	3 (0.25)	1 (0.06)	2 (0.5)	4 (0.21)	1 (0.07)	4 (0.67)	1 (1)	1 (1)	22 (0.24)	11
24	*Acacia senegal* (L.) Wild	1 (0.13)	1 (0.3)						1 (0.07)				3 (0.03)	3
3	*Acacia tortilis* (Forssk.) Hayne	4 (0.5)	2 (0.5)	2 (0.5)	8 (0.67)	6 (0.35)	4 (1)	5 (0.26)	9 (0.6)	5 (0.83)	1 (1)		46 (0.51)	10
19	*Balanites aegyptiaca* (L.) Delile				2 (0.17)	1 (0.06)		4 (0.21)	1 (0.07)	1 (0.17)			9 (0.1)	5
21	*Balanites glabra* Mildbr. & Schltr.	5 (0.63)	1 (0.3)	1 (0.25)	6 (0.5)	4 (0.24)		3 (0.16)	7 (0.47)	2 (0.33)	1 (1)	1 (1)	31 (0.34)	10
6	*Bridelia taitensis* Vatke & Pax				1 (0.08)	1 (0.06)		3 (0.16)		1 (0.17)			6 (0.07)	4
13	*Commiphora schimperi* (O.Berg) Engl.								1 (0.07)	1 (0.17)			2 (0.02)	2
17	*Cordia monoica* Roxb.				1 (0.08)			1 (0.05)					2 (0.02)	2
14	*Dalbergia melanoxylon* Guill. & Perr.				1 (0.08)	3 (0.18)		4 (0.21)	2 (0.13)	1 (0.17)		1 (1)	12 (0.13)	6
15	*Dodonaea viscosa* (L.) Jacq.	1 (0.13)			1 (0.08)				2 (0.13)				4 (0.04)	3
9	*Grewia fallax* K.Schum.	2 (0.25)	1 (0.3)			1 (0.06)			1 (0.07)	1 (0.17)			6 (0.07)	5
5	*Grewia sp.*			1 (0.25)	1 (0.08)	2 (0.12)		5 (0.26)		1 (0.17)			10 (0.11)	5
12	*Grewia villosa* Willd.							2 (0.11)	1 (0.07)	2 (0.33)			5 (0.06)	3
16	*Olea europaea ssp. cuspidata* (Wall. & G.Don) Cif.	1 (0.13)											1 (0.01)	1
7	*Opilia amentacea* Roxb.					1 (0.06)	1 (0.25)	3 (0.16)					5 (0.06)	3
1	*Ormocarpum kirkii* S.Moore	2 (0.25)	2 (0.5)	4 (1)	3 (0.25)	7 (0.41)	4 (1)	11 (0.58)	6 (0.4)	4 (0.67)			43 (0.48)	9
11	*Ozoroa insignis* Delile			1 (0.25)	2 (0.17)	2 (0.12)		1 (0.05)	3 (0.2)	1 (0.17)			10 (0.11)	6
18	*Pappea capensis* Eckl. & Zeyh.							1 (0.05)	1 (0.07)				2 (0.02)	2
10	*Rhus natalensis* Krauss					1 (0.06)		5 (0.26)	4 (0.27)				10 (0.11)	3
22	*Solanum incanum* L.									1 (0.17)			1 (0.01)	1

Frequency-i = Collection times / No. of days each informant participated. The number of days each informant participated can be found in Table 2

Frequency-t = Collection times / 91 trips (i.e. total No. of trips that the 11 girl informants undertook during the 21 days)

*Data of the types of plant species that participant N (11) collected was unable to collected during the survey

**Data concerning girls’ collection frequency of *Dombeya kirkii* Mast. and *Teclea simplicifolia* (Engl.) Verd. was not sufficiently recorded, thus I did not include these two species in this table

***No. refers to the voucher numbers that provided for plant identification

Above all, these older girls were observed to choose different species according to their personal preferences, such as hardness, smell, and color of a certain wood species. They also considered the performance of different types and conditions of wood when burning, such as fuel efficiency and the amount of smoke. Furthermore, similar with women, girls’ personal preferences differ from one girl to another.

### Interpersonal aspects of girls’ firewood collection

When collecting firewood together, girls shared their personal preferences with each other. Chl told that she didn’t like *Ormocarpum kirkii*, because its branches never become straight and are difficult to cut and pack. When Mg left some wood pieces of *Ormocarpum kirkii* for sharing with her friends, Chl and other girls took some each of them and helped her lift the heavy load onto her back; they appreciated Mg’s work. When collecting firewood together, those girls informed Sk immediately when they found dried *Rhus natalensis*, because they know she likes this wood species.

It was very rare to observe women join the girls’ groups for collecting firewood, however, girls and women share certain degrees of consensus on the perceptions of plant species usage as firewood. Table 4 shows the times and frequency with which girls gathered different wood species during the 21 days of trips listed in Table 2. The most common species collected by all the girls include *Acacia mellifera*, *Ormocarpum kirkii*, and *Acacia tortilis*. Local women have deemed these three species as “good firewood” with regard to their hardness and performance when burning (Table 1). In terms of hardness, the girls made comments similar to those of the women. For instance, Sk explained that *Acacia tortilis* is not as hard as *Ormocarpum kirkii*, and that *Acacia mellifera* is one of the most difficult to cut. She said: “The heavier and harder the wood is, the better it performs as fuel. I want the best [pieces] for my cooking.” *Acacia mellifera* and *Acacia tortilis* are easy to access and abundant in this area year round. During dry season, dry *Ormocarpum kirkii* can be also easily found in areas such as Oldonyo Wuarikon. These plant species were observed as being collected by girls of different ages the most frequently (see Table 4). They also received good reports from women. Above all, consensus among girls and women can be observed when individuals choose wood species for firewood. This probably relate to the accessibility and abundance of these species in study area.

On very few occasions the women participated in the girls’ groups, they indirectly involved themselves in girls’ firewood collection through watching attentively. In contrast with local women, teachers in the local school and tourists from other countries expressed negative comments toward firewood collection by girls as opposed to school study. For instance, some of them treat this activity as a waste of time comparing it with the time girls spend in school. In reaction, the girls wrote, “the hardest work at home is firewood collection” inside their homework. At the same time, they independently went out for firewood collection and brought back home a considerable amount of wood during the weekend. School-age girls regularly brought the firewood they collected to school for preparing school lunches. They also contributed their firewood to the teachers’ daily cooking during school days.

Local women’s positive evaluation and attention, together with interactions among peers encouraged individual girls to continue this chore. In addition, these interpersonal aspects may also have encouraged the girls to actively respond to negative comments from external sources and independently contribute to the continuation of school education through their chore participation.

## Discussion

The discussion has four foci: the personal, interpersonal, cultural-institutional aspects and their correlations related to EK acquisition by girls during firewood collection.

### Personal aspects

The independent contribution of children to the household according to external social changes can be observed in many other regions in Africa [43–45]. Children’s participation in local society through daily chores has been highly evaluated for its function in children’s identity acquisition [46]. Maasai girls independently participated in firewood collections within local gender-age roles. They intensively performed this chore during the long-term vacation before the rainy season. The load of an 11-year-old girl can be as heavy as that of adult Maasai women in Tanzania, as described by Biran and his research team [36]. Through the processes of collection, each girl developed her own sensory perceptions and preferences for different plant species with regard to the shape, smell, hardness, and weathering conditions of the wood. The evidence of difference in sensory perceptions and preferences among individual girls and women show, there are embodied personal and imparticipable aspects of EK, which can only be comprehended by individuals through independent performance of this chore along with their physical development. The personal bodily features in turn influence individual girls accessibility to different types and conditions of wood. Recent studies concerning knowledge acquisition emphasized individual’s ways of knowing as the process of acquiring knowledge within the complex, indissoluble connections among the mind, body, and environment [47, 48]. Findings of this study show, individual girls developed personal skills, perceptions and preferences for using local vegetation as firewood in different physical developmental stages, which accompany differences in individual level.

Kiringe [13] argued the differences of EK on an individual level might be a reflection of the ways that community members gradually acquire this kind of knowledge throughout their lives. Similar to Kiringe, the findings of this study shows that personal perspective itself is not enough to capture the lifelong EK acquisition of younger generations. Rather, from the autonomous and constant participation of girls in firewood collection, and their gradual acquisition of competent skills in this chore, it is clear that Maasai girls still continually acquire EK within current sociocultural and natural contexts.

### Interpersonal aspects

Only on a few occasion did local women participate in girls’ firewood collection. However, they positively supported the girls’ independent participation in this chore. For instance, the girls were allowed to go and access different types of plant species and use local tools freely. Mothers considered the health of girls rather than teach them directly about this chore. This indirect involvement of women in girls’ firewood collection enabled and encouraged the girls to independently access and practice the EK during this chore. Local adults’ positive recognition as one vital interpersonal factor constantly encouraged girls to positively evaluate their chore participation and respond to different opinions in the current social context.

From the perspective of EK acquisition, through daily interaction among girls, and between girls and adults, girls learned the diverse evaluations and perceptions of firewood collection and related plant species from other people both in and outside local community. From daily interpersonal communications, girls acquired the interpersonal aspects of EK that cannot be perceived only through individuals’ sensory experience of firewood collection. And the bodily-acquired personal EK in turn contributed to mutual understanding among peers and other members of the local community, making the EK, perceived through sensory experience during firewood collection, no longer personal but imbued with significant interpersonal and sociocultural meanings.

### Cultural-institutional aspects

Compared with previous studies, cultural-institutional change can be observed in girls’ participation in firewood collection. Grandin conducted a survey of local labor input in a Maasai village in Kenya in the 1980s. He found that girls were mostly involved in small livestock herding, and the mean time they spent on gathering wood was less than 0.2 h per day; but women collected wood for an average of 1.3 h per day [36]. Compared with his findings, girls’ firewood gathering in this study is much more time-consuming with high frequency (21 trips during 55 days) during the long-term school vacation prior to the rainy season, and this participation reduced notably during school days [42].

Several reasons for this should be considered. Firstly, local diet changes from a milk and meat centered diet to market and agriculture products dominant diet [35] had induced changes in cooking processes, which require more fuel consumption. Secondly, girls showed their motivation for guaranteeing the fuel supply in their household during school days followed local gender-age roles, but without compromising their school attendance. Girls’ EK acquisition from this point also includes the comprehension of their household strategies for coping with concurrent external social and natural environment changes.

### Persistent changing of EK acquisition within correlations of described three aspects

The above discussion shows that EK acquisition of individual girls is still continuing in concurrent social and natural contexts. At the same time, this continuation also accompanied several changes concerning concurrent personal, interpersonal and cultural-institutional factors during firewood collection. These factors are correlated to and influenced by each other, and endowed with EK concurrent multi-dimensional meanings. For instance, the EK that girls acquired through firewood collection is not only about acquiring the biological meanings of wood species, but also includes the diverse social meanings concerning peer relations, children-adult interactions during this activity, and subsistence conditions in the current social context of Maasai. It is also about girls’ active and autonomous reactions to these natural and social conditions during their participation in this chore. Ingold [28] argued that “knowing is not a matter of being in possession of information handed down from the past, but is rather indistinguishable from the life-activity of the organism-person in an environment that has itself been, and continues to be, fashioned through the activities of predecessors and contemporaries.” (pg. 302) The results of this study emphasize that Maasai girls actively perceive EK with dimensional meanings during firewood collection, and as they acquire this knowledge through daily chores, they also continuously create new meanings for the EK embedded in it.

## Conclusion

This study has examined the EK acquisition of pastoral Maasai girls. It has portrayed girls’ EK acquisition as the form of complex and adaptive empirical processes that are constantly shaped and influenced by concurrent personal, interpersonal and cultural-institutional factors during their firewood collection. The girls developed their sensory perceptions and preferences through direct interaction with different wood species during this chore. This evidence shows there are embodied personal and imparticipable aspects of EK that can only be comprehended by Maasai children through independently undertaking local chores along with their physical development. Considering the interpersonal aspect, the bodily-acquired personal EK in turn contributed to mutual understanding among peers and other members of the local community during interpersonal communication. This interpersonal aspect makes the personal bodily perceived EK no longer personal but with significant interpersonal and sociocultural meanings. Finally, from cultural-institutional aspect, Maasai girls continued their firewood collection through adjusting their participation according to local gender-age roles in labor attribution, school schedules and seasonal changes. Their EK acquisition from this point also includes the comprehension of their household strategies for coping with concurrent cultural-institutional and natural environment changes. Above all, continually experiencing these personal, interpersonal and cultural-institutional aspects in concurrent Maasai society during daily chores furnish the EK of children with multi-dimensional meanings, which enhance the adaptive nature of this knowledge system.

The findings of this study illustrate that changes in children’s participation in daily chores cannot be simply linked to their TEK acquisition as linear gain or loss of knowledge on a biological level. Rather, the TEK of children also has interpersonal and social meanings, which adaptively and continuously change for coping with external social and natural environment complexities. Changes in children’s chore participation may not always induce the reduction of their TEK, rather, it may contribute to the creation of new meanings of TEK in concurrent social contexts. Further studies need to pay more attention to the changes in children’s daily activities, and explore the TEK acquisition of children while considering their contribution to the concurrent multi-dimensional and adaptive meanings of this knowledge system.

## Abbreviations

EK: Ethnobotanical knowledge
KGR: Kuku Group Ranch
TEK: Traditional Ecological Knowledge

## Footnotes

1 I use ‘females’ to present both girls and adult females, and ‘women’ to indicate only adult females in this paper.

2 A household in this study refers to a family unit where members live together in the same house. The Maasai have a polygamous system, and each wife of a man builds her own house. They live with and cook for their own children, and the husband chooses which house to eat in. Firewood consumption is associated with family members who live in the same house.
